# Sepsis Unveils T-cell Large Granular Lymphocytic Leukemia in the Setting of End-Stage Renal Disease: A Rare Hematologic Malignancy

**DOI:** 10.7759/cureus.55325

**Published:** 2024-03-01

**Authors:** Tutul Chowdhury, Kalendra Kunwar, Fareeza Mustafa, Annmarie T Sajeev, Mrinal Sharma, Muhammad N Pasha, Madhumati Kalavar

**Affiliations:** 1 Internal Medicine, One Brooklyn Health Interfaith Medical Center, New York, USA; 2 College of Medicine, American University of Antigua, Jabberwock, ATG; 3 Pulmonary and Critical Care Medicine, One Brooklyn Health Interfaith Medical Center, New York, USA; 4 Hematology and Oncology, One Brooklyn Health Interfaith Medical Center, New York, USA

**Keywords:** methicillin-sensitive staphylococcus aureus, flow cytometry, neutropenia, sepsis, leukemia

## Abstract

Large granular lymphocytic (LGL) leukemia is a rare chronic lymphoproliferative disorder originating from natural killer cells or T lymphocytes. In this report, we present the case of a 66-year-old female initially treated for sepsis, with* *methicillin-sensitive* Staphylococcus aureus* identified on initial blood culture prompting intravenous (IV) antibiotic therapy. The patient met systemic inflammatory response syndrome criteria upon admission due to severe neutropenia. Persistent fever led to neurological symptoms, and imaging revealed lung abnormalities along with chronic changes on the CT scan of the head. Multidisciplinary consultations were sought, resulting in treatment adjustments including antifungals and filgrastim. Flow cytometry and bone marrow biopsy confirmed the diagnosis of LGL leukemia.

## Introduction

Individuals diagnosed with T-cell large granular lymphocytic (LGL) leukemia usually exhibit neutropenia, autoimmune diseases, and recurrent bacterial infections in their sixth decade of life [1]. The clonal proliferation of cytotoxic T cells or natural killer (NK) cells is a hallmark of LGL, an uncommon and indolent hematologic malignancy. Notwithstanding its rarity, LGL leukemia poses a substantial clinical challenge because of its diverse appearance and unpredictable outcome [2]. Patients frequently exhibit nonspecific symptoms such as weariness, cytopenia, and recurring infections, complicating diagnosis and treatment. Although LGL leukemia was first identified as a chronic lymphoproliferative illness of mature T lymphocytes or NK cells, new developments in molecular techniques have illuminated the pathophysiology of the disease and suggested treatment possibilities. In this case report, we present the clinical course, diagnostic challenges, and management strategies in a patient diagnosed with LGL leukemia, highlighting the importance of a multidisciplinary approach in optimizing patient outcomes.

## Case presentation

A 66-year-old female patient with a past medical history of end-stage-renal-disease (ESRD) on hemodialysis, neutropenia, combined systolic and diastolic heart failure, normocytic normochromic anemia, and hypertension presented to the emergency department for generalized weakness, persistent cough, weight loss, loss of appetite, and loose stool for more than a month. She denied fever, chills, and shortness of breath on presentation. The patient had a similar presentation one month ago prior to this admission, with fever, chills, and fatigue. On earlier admission, blood culture revealed methicillin-susceptible *Staphylococcus aureus* thus, she was treated with intravenous (IV) antibiotics (cefazolin). The patient again returned to the emergency department two weeks ago with persistent fever, palpitations, and fatigue. She was managed with IV antibiotics (ceftriaxone, vancomycin, and cefepime), filgrastim, and erythropoietin. The patient was scheduled for a bone marrow biopsy, but she left the hospital against medical advice.

On presentation this time, vitals showed blood pressure of 92/53 mm of Hg, heart rate 106 beats per minute, respiratory rate 17 per minute, and temperature 98.7 degrees Fahrenheit (F), and physical examination was unremarkable. The patient met the systemic inflammatory response syndrome criteria on admission. Laboratory investigations revealed white blood cells of 0.4 10x3/uL with absolute neutrophils of 0.0 10x3/uL, absolute lymphocytes of 0.3 10x3/uL, red blood cells of 3.53 10x6/uL, hemoglobin 10.1 g/dL, and platelets 131 10x3/uL (as shown in Table 1).

**Table 1:** Labs done on admission and two weeks after admission IgM: Immunoglobulin M

Test	Ref range and units	Values (on admission)	Values (two weeks after admission)
White blood cell	4.5-11.0 10x3/uL	0.5 10x3/uL (L)	0.4 10x3/uL (L)
Hemoglobin	11.0-15.0 g/dL	9.2 g/dL (L)	10.1 g/dL (L)
Mean corpuscular volume	80-100 fL	93.6 fL	91.1 fL
Platelets	130-400 10x3/uL	175 10x3/uL	131 10x3/uL
Blood urea nitrogen	7.0-18.7 mg/dL	26 mg/dL (H)	20 mg/dL (H)
Creatinine	0.57-1.11 mg/dL	9.3 mg/dL (H)	6.5 mg/dL (H)
Estimated glomerular filtration rate	>=90.0	4.3 (L)	6.6 (L)
Sodium (Na)	136-145 mmol/L	141 mmol/L	138 mmol/L
Potassium (K)	3.5-5.1 mmol/L	3.9 mmol/L	19.70 mmol/L (H)
Bicarbonate	22-29 mmol/L	24 mmol/L	25 mmol/L
Anion gap	mmol/L	13 mmol/L (H)	16mmol/L (H)
Total bilirubin	0.2-1.2 mg/dL	0.7 mg/dL	0.5 mg/dL
Alanine aminotransferase	10-55 U/L	<3 U/L (L)	13 U/L
Aspartate aminotransferase	5-34 U/L	12 U/L (L)	104 U/L (H)
Alkaline phosphatase	40-150 U/L	58 U/L	58 U/L
Albumin	3.5-5.2 g/dL	3.5 g/dL	3.6 g/dL
Calcium	8.4-10.2 mg/dL	8.5 mg/dL	8.9 mg/dL
Brain natriuretic peptide	10-100 pg/ml	2166 pg/ml (H)	1391 pg/ml (H)
Lactate	0.50-1.90 mmol/L	1.4 mmol/L	1.9 mmol/L
High sensitivity troponin	0-17 ng/ml	219.9 ng/ml	Not done
Prothrombin time	9.8-13.4 sec	12.3 sec	11.0
International normalized ratio	0.85-1.15	1.03	0.93
Partial thromboplastin time	24.9-35.9 sec	32.4 sec	34.5
Procalcitonin	0.00-0.08 ng/ml	0.58 ng/ml (H)	5.75 ng/ml (H)
Hemoglobin A1c	4.8-5.6%	5.10%	5.10%
Reticulocyte percent	0.5-2%	1.89%	0.60%
Ferritin	17.90-464.00	1262.80 (H)	3289.00 (H)
Hepatitis B surface antigen	Negative	Non-reactive	Not performed
Hepatitis B core IgM	Negative	Non-reactive	Not performed
Hepatitis C	Negative	Non-reactive	Not performed
Hepatitis A antibody, IgM	Negative	Non-reactive	Not performed

**Figure 1:**
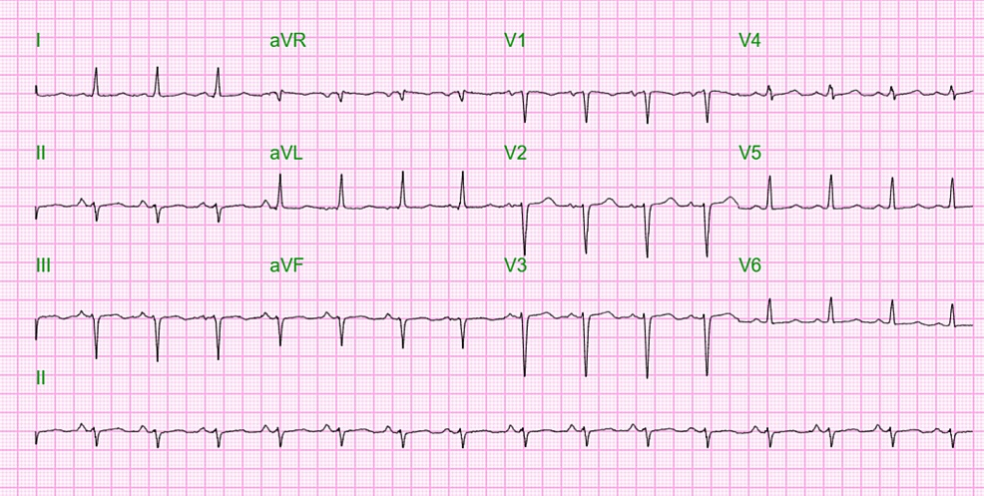
Electrocardiogram showing normal sinus rhythm

An electrocardiogram showed normal sinus rhythm (as shown in Figure 1).

**Figure 2:**
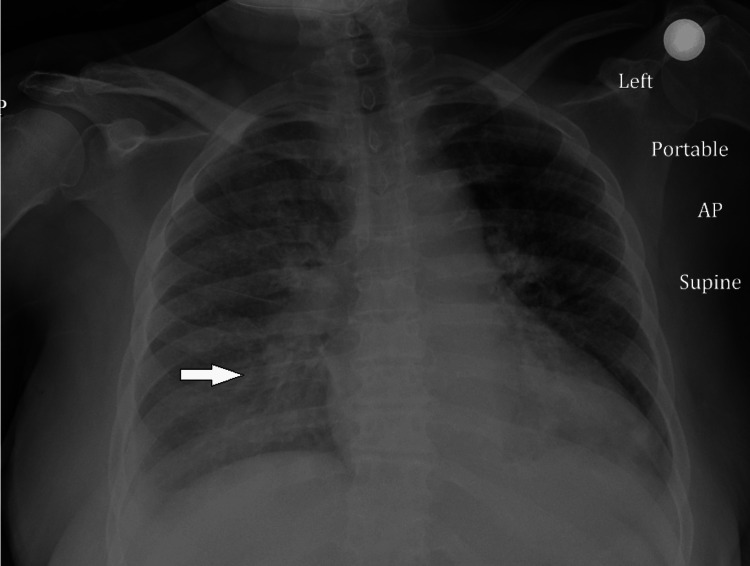
Chest X-ray showing lung infiltrates (white arrow)

Chest X-ray showed right-sided lung infiltrates (as shown in Figure 2).

**Figure 3:**
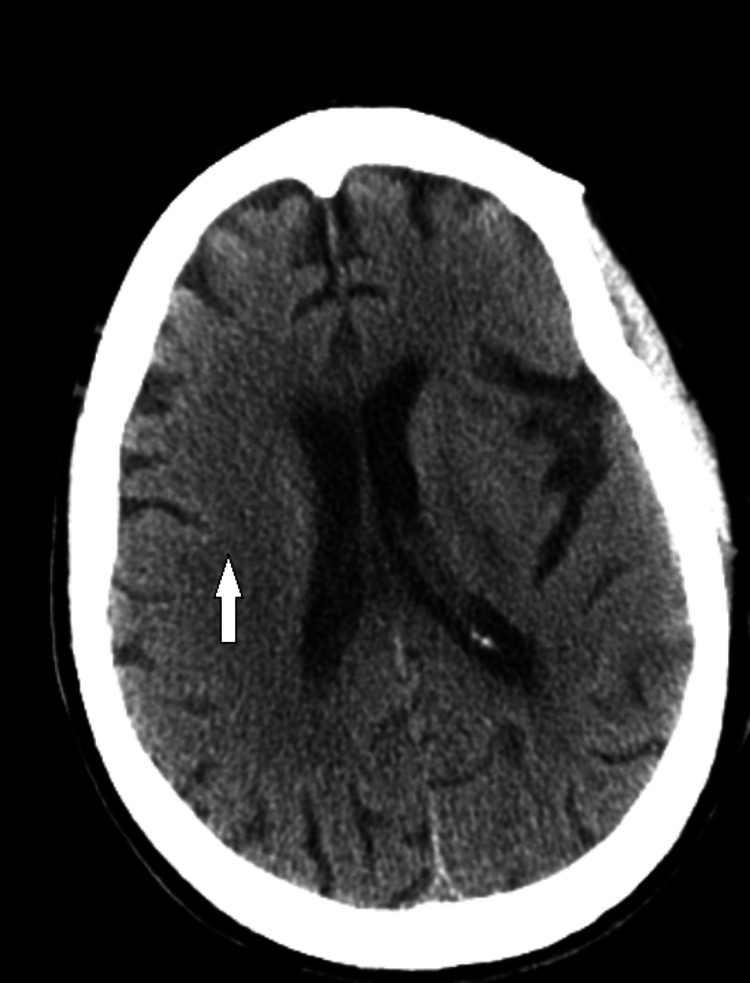
Computed tomography (CT) scan of the head showed moderate diffuse chronic senescent changes (white arrow)

The patient was managed with a stat dose of IV vancomycin, cefepime, fluconazole, and metronidazole on presentation. The patient spiked fever the next morning with the highest temperature reaching 104 F with a change in her mental status. A computed tomography (CT) scan of the head showed moderate diffuse chronic senescent changes (as shown in Figure 3).

**Figure 4:**
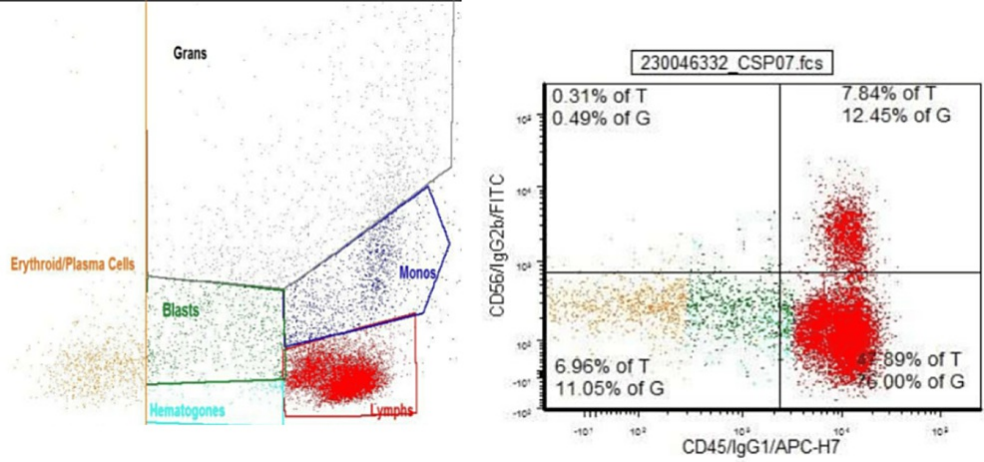
Flow cytometry suggestive of large granular lymphocytic (LGL) leukemia Grans: Granulocyte; Blasts: Blast Cell; Monos: Monocyte; Lymphs: Lymphocyte; T: T Lymphocyte; CD: Cluster of differentiation

Cefepime and metronidazole were discontinued on day two and started on meropenem with continuation of fluconazole, and vancomycin. Despite broad-spectrum antibiotics coverage, she continued to spike fever, thus hematology was consulted and was started on filgrastim on day two. Valacyclovir was added for suspected herpes infection. Herpes simplex virus 1/2 polymerase chain reaction (PCR) was reported negative and valacyclovir was discontinued. A wound culture from the vulva revealed *Pseudomonas aeruginosa* and gentamicin was added to the regimen. The stool for clostridium difficile PCR, blood, and urine culture were negative. Infectious disease specialists were consulted for persistent altered mental status and acyclovir was initiated on day seven. The patient and her next of kin refused the lumbar puncture. CT chest repeated on day eight resulted in multifocal ill-defined alveolar infiltrates. Furthermore, the patient was minimally communicative, lethargic, and disoriented on day eight and voriconazole was started for suspected pulmonary aspergillosis. Flow cytometry reported lack of CD16 and CD10 in neutrophils, aberrant expression of CD56 and CD23 in monocytes. In addition, analysis of the leukocyte population showed granulocytes 19%, monocytes 4%, lymphocytes 76%, blasts 1%, B cells 3%, T cells 69%, LGLs 19%, and NK cells 4%. Bone marrow biopsy was performed, and flow cytometry showed (as shown in Figure 4) abnormal myeloid maturation on myeloid elements with relatively increased (1.5 % of total) myeloblasts as well as relatively increased T lymphocytes (42.3% of total) with especially nature killer large granular lymphocytes (7.9% of total), but without immunophenotypic evidence of clonal B-cell population; concluded as LGL leukemia.

T-cell gene rearrangement PCR and chromosome analysis were sent. The patient was discharged to another facility for further management.

## Discussion

Leukemia, traditionally perceived as one of the most prevalent and manageable cancers with favorable prognoses, assumes a chronic and asymptomatic course when categorized as a subtype of LGL leukemia. Demographic studies conducted in Europe and America estimate the average incidence of this rare cancer to be between 0.2 and 0.72 cases per million persons annually [1,3]. LGL leukemia is characterized by the clonal proliferation of large granular lymphocytes derived from either NK cells or T cells. While NK cells function as components of innate immunity, T cells operate within the adaptive immune system, identifying specific antigens and mounting targeted immune responses. Within the realm of LGL leukemia, NK cell involvement may further manifest as a signal transducer and activator of transcription 3 (STAT3) mutated NK cell leukemia, presenting as an aggressive form with early systemic disease and organomegaly [2,3]. Conversely, T-cell leukemia, purportedly activated by specific antigen-receptor binding, presents as a more chronic and indolent variant. The association of T-cell LGL leukemia with multiple comorbidities and autoimmune diseases, along with the subsequent immunosuppressive treatments administered to affected patients, may obscure an increasing prevalence within American populations.

T-cell LGL is diagnosed as persistent clonal proliferation of memory-effective T cells in the peripheral blood that lasts for months and subsequent infiltration into the bone marrow, spleen, and liver. The activation of T cells by antigens results in clonal proliferation of mature post-thymic T lymphocytes, which is a natural immune response. However, in LGL leukemia, the polyclonal proliferation mutates and leads to increased cytokines, granzymes, and cytotoxicity. Interleukin 15 and platelet-derived growth factor have been distinguished as cytokines that dysregulate the Janus Kinase-signal transducer and activator of transcription proteins (JAK-STAT) and MAPK (mitogen-activated protein kinase) pathways and stimulate anti-apoptotic proteins and decrease the production of pro-apoptotic proteins. Eventually, the mutations lead to resistance to activation-induced cell death, keeping them alive for a longer period [1,4,5]. T-cell leukemia is diagnosed through PCR which identifies TCR that are the result of mutations [2].

Symptoms of T-cell leukemia vary from patient to patient with several of them developing hematological and autoimmune states. In a study done in 1994, the most common manifestations of T-cell LGL were constitutional, with 60% of patients expressing fatigue [3]. The most common hematological complication was neutropenia with 50% of patients diagnosed with absolute neutropenia, and 10-15% of patients reported at the time of diagnosis to have infections or a history of infections [3] as in the patient described in this report. However, several other autoimmune conditions were also found in patients. The heterogeneity of symptoms can result in T-cell LGL, a difficult diagnosis to conclude. Therefore, it is important to keep T-cell LGL as a differential when diagnosing patients with long-term inflammatory conditions. As per research, it seems this is one of the rarely reported cases of T-cell LGL in the setting of ESRD. T-cell LGL remains a cancer known for its asymptomatic course, and treatment is supportive of its symptoms. In a study conducted in France, 23 of the patients who were observed to develop kidney injury were treated with corticosteroids and several lines of immunosuppressive regimens including cyclosporine, methotrexate, tumor necrosis factor alpha inhibitors, and leflunomide [5]. The association of T-cell LGL with several autoimmune diseases, including the diseases identified in the kidney, provided a reason for immunosuppression [3]. However, the result of immunosuppressive treatment led to no detection of clonality.

In addition, the JAK inhibitor tofacitinib was utilized in some of the patients. In the same study population, identification of the JAK/STAT pathway as a contributor to inflammatory fibrosis was observed due to phospho-STAT3 staining in the kidney samples of six patients [4]. The staining was positive in lymphocytes, renal tubules, and endothelium. The staining also turned positive in some of the patient’s liver samples indicating inflammatory pathology. One patient who had interstitial nephropathy and was refractory to other immunosuppressive regimens was treated with tofacitinib. He demonstrated dramatic improvement which led to complete remission of thrombocytopenia and vitiligo, but kidney function stabilization [3,5,6]. T-cell LGL treatment is exploratory and needs to be further researched for adverse effects and long-term events. More commonly, T-cell LGL is treated in symptomatic patients and immunosuppressive therapy is the standard treatment with adjuvant treatment for the autoimmune diseases the patient may have [7,8]. T-cell LGL continues to be an atypical leukemia that often remains undetected.

## Conclusions

In conclusion, LGL leukemia represents a rare subtype of leukemia characterized by the clonal proliferation of large granular lymphocytes derived from either NK cells or T cells. While traditionally perceived as manageable, LGL leukemia presents unique challenges due to its chronic and asymptomatic nature, often leading to delayed diagnosis. Despite its rarity, associations with multiple comorbidities and autoimmune diseases suggest a potential underestimation of its prevalence. Diagnostic methods such as PCR play a crucial role in identifying T-cell LGL, which manifests with a range of symptoms including hematological complications and autoimmune states. Treatment approaches, while predominantly supportive, may include immunosuppressive regimens and novel agents targeting specific signaling pathways. Further research is needed to better understand the pathophysiology of T-cell LGL and explore potential therapeutic interventions for improved patient outcomes.
